# Outcomes for Women Denied Postpartum Tubal Ligation During the Initial COVID-19 Surge

**DOI:** 10.1089/whr.2023.0142

**Published:** 2024-04-23

**Authors:** Lauren Cosgriff, Melissa Plummer, Gabrielle Concepcion, Antoinette A. Danvers

**Affiliations:** ^1^ Department of Obstetrics, Gynecology and Women's Health, Albert Einstein College of Medicine/Montefiore Medical Center, Bronx, New York, USA.; ^2^ Department of Obstetrics and Gynecology, Brigham and Women's Hospital, Boston, Massachusetts, USA.; ^3^ Department of Obstetric and Gynecology, New York University School of Medicine, New York, New York, USA.

**Keywords:** postpartum sterilization, postpartum long-acting reversible contraception, contraception, COVID-19

## Abstract

**Objective:**

To evaluate the utilization and outcomes of postpartum long-acting reversible contraception (PPLARC) following unmet postpartum bilateral tubal ligation (PPBTL) requests during a time in which elective surgeries were canceled due to the initial COVID-19 surge.

**Methods:**

We conducted a mixed-methods study using an embedded design. Using a retrospective cohort design, we collected data from patients seeking PPBTL following vaginal delivery between March 15, 2020, and June 20, 2020; this reflects a time period during which elective surgery was canceled thus making PPBTL unavailable. We recorded demographic data, method of contraception at time of discharge and 18 months postpartum, and incidence of interval pregnancy at 18 months postpartum. Additionally, we conducted five semistructured interviews to gain deeper insights into patient experiences with PPLARC as a bridge method.

**Results:**

Forty-five patients had unfilled PPBTL requests with follow-up data available for 35. The median age was 34 years. Ten (22%) accepted PPLARC as a bridge to interval bilateral tubal ligation (BTL). At the 18-month mark, only 1 out of 7 (14.3%) PPLARC users had undergone an interval BTL procedure, compared to 11 out of 28 (39.3%) nonusers. None of the PPLARC users experienced pregnancies, while 6 out of 28 (21.6%) nonusers became pregnant. Qualitative interviews underscored themes such as inadequate counseling preparation for unmet PPBTL requests and persistent barriers to BTL access.

**Conclusions:**

Raising awareness of unmet PPBTL risks may drive greater adoption of PPLARC as a bridge method. While not a substitution for PPTBL, PPLARC provides a reliable form of interval contraception for patients seeking to delay pregnancy. It is essential to recognize that patient security with PPLARC's contraceptive efficacy may introduce delays in achieving the desired interval sterilization. Enhancing antenatal counseling on contraception options and providing transparency regarding barriers to sterilization could mitigate the challenges associated with unmet PPBTL requests.

## Introduction

Tubal sterilization, also referred to as permanent contraception, is a highly effective contraceptive method with a failure rate of 0.5%. It remains the method of choice by 18% of contraceptive users.^1,2^ The postpartum period has long been recognized as an opportune time for sterilization procedures, yet nearly 50% of tubal sterilizations go unfulfilled.^3^

Hospitals have faced persistent barriers to completing postpartum sterilization procedures, with the most prevalent hurdles including issues with obtaining valid consent forms, particularly the mandated state-required consents for publicly insured patients, limited operating room availability, and considerations related to patient factors such as maternal obesity.^3–5^ Furthermore, patients with unfulfilled tubal ligation request are at increased risk for subsequent unintended pregnancies and short interval pregnancies. A study of high-risk patients who were unable to obtain postpartum tubal sterilization found that 17% experienced a subsequent pregnancy during a 27-month follow-up period.^6^ Addressing these challenges requires concerted efforts to improve sterilization fulfillment rates and the provision of reliable alternatives as bridges to interval sterilization. These efforts are crucial in mitigating the incidence of unintended pregnancies that often stem from unfulfilled bilateral tubal ligation (BTL) requests.

The COVID-19 pandemic presented an extraordinary scenario. During the first wave of the pandemic, hospitals in New York City canceled all elective surgeries including postpartum bilateral tubal ligation (PPBTL). We expected that not completing any PPBTL during the pandemic would exacerbate an already fraught problem of PPBTL fulfillment. The objective of this study was to describe how access to PPBTL changed during the initial wave of the COVID-19 pandemic, evaluate the adoption of immediate postpartum long-acting reversible contraception (PPLARC) methods during this time, and assess the 18-month outcomes in cases where PPBTL requests remained unfulfilled. We also aimed to gain a comprehensive understanding of patients' experiences with PPLARC as a bridge method to interval BTL.

## Materials and Methods

The coronavirus pandemic introduced new barriers to fulfilling postpartum tubal ligation (PPBTL) requests. New York City was the epicenter of the outbreak in the first wave. In response to an expected surge in cases, hospitals closed most outpatient clinical sites and canceled elective surgeries. Our institution implemented these changes on March 15, 2020. Tubal sterilization procedures, including PPBTL, were considered elective procedures and thus unable to be performed. We employed a mixed-methods study with an embedded design. We conducted a retrospective observational study to investigate the impact of the initial COVID-19 surge and elective surgery ban on PPLARC utilization as a bridge to interval BTL and its outcomes. The study focused on patients who expressed a desire for PPBTL during this time. In parallel, we conducted semistructured interviews with patients who opted to receive a PPLARC methods to explore their experience with PPLARC as a bridge method.

The study was conducted at Montefiore Medical Center, a tertiary care medical center located in Bronx, New York. The center serves a diverse patient population and offers a range of reproductive health care services. The Bronx was one of the areas that experienced a surge in COVID-19-related hospitalizations during this period.

The study population consisted of patients who had a vaginal delivery and had a documented plan for PPBTL between March 15, 2020, and June 20, 2020, identified through a comprehensive review of electronic health records (EHR). Patients were included in the study if they had documentation of the PPBTL plan within prenatal or labor and delivery records, further corroborated by the completion of the Medicaid Title XIX form. Upon admission, these patients were informed that PPBTL would be deferred indefinitely, and those without contraindications were offered an alternative method of contraception including immediate PPLARC. Immediate long-acting reversible contraception (LARC) was previously available for postpartum placement at our institution.

For the quantitative component, we collected demographic data including age, race, and pregnancy history, contraception plan, the receipt of PPBTL, and choice of contraception at hospital discharge. We assessed contraception use, interval BTL rates, and interval pregnancy at 12- to 18-month postpartum. Follow-up data were obtained through EHR reviews and, when necessary, via phone calls to patients to ensure comprehensive data collection. The data were recorded using a REDCap software specifically created for this study.

Descriptive statistics were employed to summarize the study cohort's demographic characteristics. The proportion of patients who selected PPLARC and alternative methods used at discharge was determined. We also described the types of contraception used at 18 months, proportion receiving an interval BTL, and any interval pregnancy. The statistical analysis was conducted using STATA 17.0 (College Station, TX, USA).

To complement the quantitative findings, we conducted qualitative interviews with a limited subset of participants (*n* = 5). While the qualitative sample size was small due to the small number of patients meeting inclusion criteria, it was deemed sufficient for a supplementary exploration of participants' experiences and perspectives regarding PPBTL and contraception choices during the COVID-19 pandemic. We conducted five semistructured interviews with patients who received a PPLARC. Interviews were audio-recorded and transcribed verbatim. Thematic analysis was employed by the research team (A.D. and L.C.) who independently reviewed the transcripts to identify key themes related to counseling experiences, contraception choices, and access to PPBTL.

The study was conducted following approval by the Institutional Review Board at Albert Einstein College of Medicine.

## Results

Demographic characteristics at described in Table 1. The median age at presentation was 34 years for both groups, with a median parity of two. The majority of patients identified as Hispanic (68.6%) and Black (24.4%). Counseling regarding LARC was provided to patients during prenatal appointments or upon presentation to labor and delivery for 62.2% of patients; 22.2% accepted PPLARC as a bridge.

**Table 1.** Patient Characteristics (*n* = 45)

Characteristics	Median or ***n***	Range or %
Age	34	24–42
Hispanic	31	68.9
Black	11	24.4
Gravidity	5	2–14
Parity	2	1–7
Contraception at discharge		
BTL	0	0
IUD	1	2.2
Implant	9	20
Medroxyprogesterone acetate	4	8.9
Progesterone only pill	12	26.7
None	14	31.1

BTL, bilateral tubal ligation; IUD, intrauterine device.

**FIG. 1.**
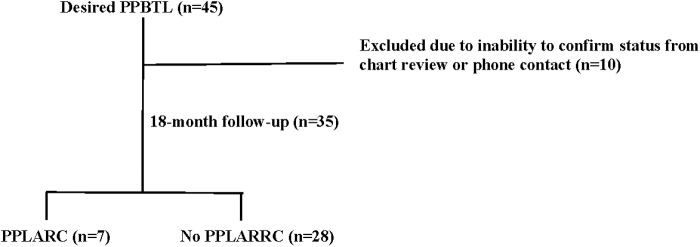
Flow of chart review. PPBTL, postpartum bilateral tubal ligation; PPLARC, postpartum long-acting reversible contraception.

Among the patients with unfilled PPBTL requests, 35/45 (77.8%) had follow-up data regarding contraception use at 18-month postpartum (Figure 1). Among them, 12 patients (34%) had received an interval BTL, 17 (49%) were still awaiting the procedure, and 6 (17%) no longer desired that method. Of the 17 who were still desiring of awaiting the procedure, 5 had received a PPLARC at delivery and had continued that method as a bridge.

**FIG. 2.**
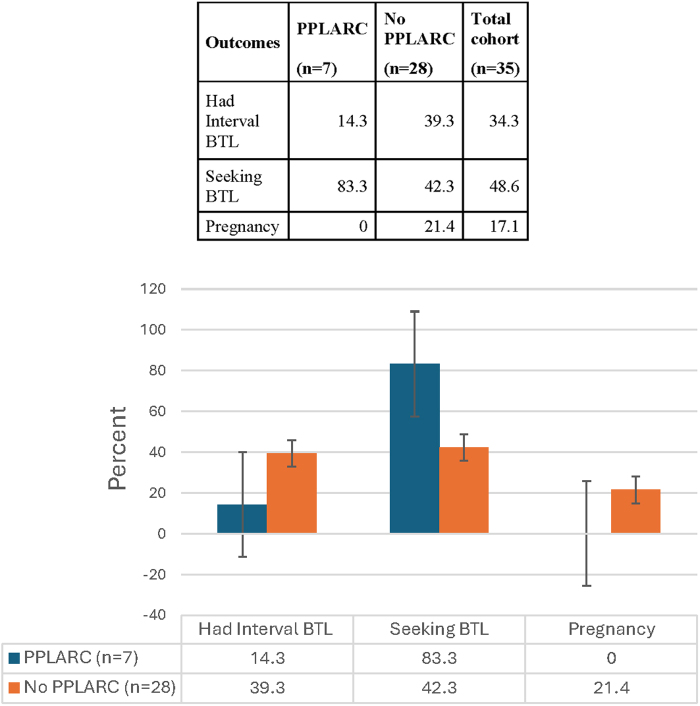
Outcomes up to 18-month postpartum after unfulfilled PPBTL by acceptance of PPPLARC as a bridge. BTL, bilateral tubal ligation.

Among patients discharged with a LARC method (*n* = 7), none had a pregnancy by 18 months. In contrast, among patients discharged with other contraceptive methods, 6/28 (21.1%) experienced pregnancies by 18-month postpartum (Figure 2).

In the qualitative component of the study, thematic analysis of interviews with a subset of participants (*n* = 5) revealed three main themes (Table 2). First, participants expressed that counseling did not adequately prepare them for the unavailability of PPBTL, with some feeling deceived by the lack of prior information.

**Table 2.** Summary of Main Thematic Findings from the Qualitative Interviews

Theme	Subtheme	Patient quotes
Counseling	Surprised at inability to receive PPBTL	“No one told me that it would not be possible. I went to the classes, I went to everything they told me, and on the day of the delivery they told me that it would not be done.” “[T]hey didn't tell me in the moment, they told me after I had recovered and was waiting for the ligation…It won't happen because the operating rooms are closed.” “[T]hey didn't give me a greater explanation, they only told me that it was because of the pandemic, and I understood that. Because it was a very difficult time”
Choice	Limited contraception choices	“So, I keep waiting to see if it can be done. But I sort of no longer have the desire to go again, because even if I say, “I want this,” it doesn't happen.” “Yes, because they say they give you an option, right? But in reality, they don't allow you decide for your own body.” “[T]hey couldn't do it at the moment, that the only option, that we would have to wait, was the injection or implant. They gave me the implant, and then I told them that I wasn't sure about the implant because I didn't feel comfortable with it…it wasn't what I want, I think it was what they thought was good for me, but for me, I was not satisfied.”
	Time to make a new decision	“I feel like I had enough time to make a decision. It wasn't a rush—I wasn't pushed towards getting on birth control or anything like that. She just gave me the information I needed, told me to take my time” “[T]hey told me that if I gave birth normally, they won't be able to sterilize me right away. But I was supposedly requesting the c-section because of this, so that I could be sterilized right away. But the decision I made was better, which was to give birth normally, I left satisfied, I recovered more quickly, and I left with my little gadget”
Barriers to access PPBTL	Persistent beyond hospitalization	“[I]t may not occur because of x reason, but afterwards you can schedule an appointment, or someone will contact you, and in reality, they trick you, they get your hopes up that they're going to do it, but they don't do it. That's the worst.” “I then called and they gave me the appointment and I went …but at that time my husband was about to start at another job where we would have to leave home for six months. So, I didn't have anyone to help me post-surgery.”
	PPLARC provided security to delay surgery	“It's four kids and I wasn't going to have anyone to help me at home, which is why I told the doctor that it was better for me to get the IUD and that I would postpone the ligation” “I'll decide then cause right now I'm fine with my birth control. I'm not having any issues with it, and it isn't bothering me. So, I figure, whenever I have to take it out, then I would just talk about it with my doctor again.”

PPBTL, postpartum bilateral tubal ligation; PPLARC, postpartum long-acting reversible contraception.

“No one told me that it would not be possible…the day of the delivery they told me that it would not be done.”

“[T]hey didn't tell me in the moment, they told me after I had recovered…he says, “No. It won't happen because the operating rooms are closed.”

Second, although all participants were presented with contraception choices, they had variable experiences with this counseling. One patient felt that they did not truly have a choice and that decisions were made for them through lack of access, while another felt the counseling they received allowed them to think about another choice.

“Yes, because they say they give you an option, right? But in reality, they don't allow you decide for your own body.”

“I feel like I had enough time to make a decision. It wasn't a rush—I wasn't pushed towards getting on birth control or anything like that. She just gave me the information I needed, told me to take my time.”

Finally, participants discussed barriers to accessing PPBTL beyond the postpartum period, both due to health system barriers or patient barriers. They voiced that PPLARC had allowed them to safely defer sterilization.

“[They say] afterwards you can schedule an appointment, or someone will contact you, and in reality, they trick you, they get your hopes up that they're going to do it, but they don't do it.”

“I then called and they gave me the appointment and I went …but at that time my husband was about to start at another job where we would have to leave home for six months. So, I didn't have anyone to help me post-surgery.”

“I'll decide then cause right now I'm fine with my birth control. I'm not having any issues with it, and it isn't bothering me.”

## Discussion

Our study addresses an important issue faced by patients seeking postpartum sterilization: A desire for highly effective contraception. Historically, a significant proportion of these patient encountered unfulfilled tubal ligation requests, with estimates ranging from 30% to 50%.^7,8^

Patients with unfulfilled tubal requests who still desire sterilization after discharge must wait for interval BTL typically performed after 6-week postpartum. Some patients face many obstacles returning for interval BTL and up to 50% will become pregnant within 12 months.^9–11^ Only 34% of the patients in this study underwent interval BTL and 17% experienced a short interpregnancy interval. The complexities and delays associated with obtaining interval BTL have been well-documented in the literature.^4,5,7^ Our findings echo the challenges patients faced pre-COVID and extended beyond the lockdown period due to cancelations resulting from positive COVID-19 screening.^12^ Patients face other multifaceted barriers, ranging from limited operating room availability to personal circumstances that hinder their ability to access timely interval BTL. This was also revealed in our interviews whereby patients describe barriers to scheduling, navigating their return home, resuming familial responsibilities, and navigating work.

Amidst these challenges, PPLARC emerges as a compelling solution to address patients' immediate contraceptive needs, substantially diminishing the risk of unintended pregnancies. LARC offers similar efficacy as PPBTL^1^ and is a good substitute for some patients seeking sterilization. However, it is important to recognize that while PPLARC offers a viable alternative to PPBTL, it may potentially delay patients' fulfillment of their desired interval sterilization. This may occur due to security with the PPLARC's ability to prevent pregnancy once barriers to interval BTL access are encountered.

Our qualitative interviews revealed that patients are particularly vulnerable when they are surprised that desired PPBTL was not available. They reported receiving information about the procedure, but not about the potential barriers posed by the pandemic until they presented for delivery. While patients were offered contraception choices, they felt that they didn't have a genuine choice. Because of the lack of preparations, some believed that decisions were made for them rather than with them, leading to dissatisfaction with their chosen method. On a positive note, some patients felt that counseling provided them with enough time to make informed decisions, without feeling rushed into choosing a contraceptive method.

A previous study showed that initiation of bridge contraception before obtaining interval LARC and sterilization was associated with increased interval LARC and sterilization fulfillment.^11^ In our study, however, patients with PPLARC as a bridge were less likely to receive interval BTL despite ongoing desire. Again, for two of the patients interviewed, the PPLARC allowed they to continue to delay plan for PPBTL as they felt secure with the method.

This study was conducted at a single medical center with a small sample size, limiting generalizability to other health care settings. The small sample size for the qualitative component may limit the transferability of the qualitative findings. There is opportunity to improve the generalizability of the study by collecting data from this same timeframe at other institutions or to compare the collected data to pre-pandemic rates of unfulfilled PPBTL requests. We may additionally seek to expand our qualitative results by performing interviews with those patients who declined PPLARC as a bridge method.

Despite these limitations, we were able to obtain follow-up data through chart review or phone call for 78% of the cohort. The mixed-methods approach, while limited in sample size, allowed for a comprehensive exploration of the challenges faced by patients seeking postpartum sterilization and provided valuable insights into patients' experiences and perspectives on how they viewed the options presented to them. Furthermore, our study reflects the real-world impact of the COVID-19 pandemic on reproductive health care practices.

Our study sheds light on an area where improvement is urgently needed. The data underscore the importance of enhancing access to both PPBTL and interval BTL. Such improvements should both focus on reducing wait times and streamlining the interval BTL process and enhance patient counseling about contraception options as bridge methods. These efforts should be guided by the goal of empowering women to make informed decisions about their reproductive health while ensuring timely access to the contraceptive method that aligns with their preferences and life circumstances.

## Abbreviations Used

BTL: bilateral tubal ligation
EHR: electronic health record
IUD: intrauterine device
LARC: long-acting reversible contraception
PPBTL: postpartum bilateral tubal ligation
PPLARC: postpartum long-acting reversible contraception
